# The Influence of Managerial Psychology on Job Satisfaction among Healthcare Employees in Ghana

**DOI:** 10.3390/healthcare8030262

**Published:** 2020-08-11

**Authors:** Baozhen Dai, Benedicta Akey-Torku

**Affiliations:** School of Management, Jiangsu University, 301 Xuefu Road, Zhenjiang 212013, China; hixiaodai@126.com

**Keywords:** job autonomy, psychological capital, job satisfaction, healthcare professionals

## Abstract

**Background:** Employee job satisfaction has been established to be one of the important factors that work towards addressing the subject matter of productivity in organizations. Healthcare professionals deserve some level of basic psychological need satisfaction in the area of job autonomy. Reasons that lead to employees achieving job autonomy and job satisfaction have been researched by industrial and organizational psychologists but very few of such studies have directed their attention towards the role psychological capital can play. Therefore, this study sought to find out how much of an impact positive psychology can make on the job autonomy of healthcare employees leading to the fulfillment of job satisfaction. **Methods:** Data were collected from 385 healthcare professionals from the public sector. A structural equation model was performed to analyze the relationship that exists between the constructs of psychological capital and job autonomy leading to job satisfaction on the part of the employees. **Results:** Results showed both a direct and indirect positive relationship between hope and job satisfaction and indirect through job autonomy. Apart from self-efficacy, that had a very low positive relationship, optimism largely influenced job autonomy of healthcare professionals. Results also showed that psychological capital positively related to job autonomy while job autonomy minimally influenced job satisfaction. **Conclusions:** It is concluded from this study that healthcare professionals deserve some level of basic psychological need satisfaction in the area of job autonomy and that can stimulate positive work ethic.

## 1. Introduction

A hospital is an important engine for social progress. For this reason it must constantly change its operational strategy to accommodate new ideas and challenges that faces society. A successful modern hospital makes effort to attract and retain highly skilled clinical and non-clinical staff since effective, quality and reliable health service delivery depends on high quality, committed, and emotionally and psychologically sound workers. The effect of the 2019 novel coronavirus disease (COVID-19) on hospital personnel in recent times testifies to the importance of health professionals in contemporary hospitals. As stated in Xu et al. [1], even though the history of modern medical practice documents overwhelming concerns about the welfare, satisfaction and commitment of health professionals, the emergence of COVID-19 has exacerbated this concern. All over the world, frontline workers have been overstretched as they navigate healthcare pathways to ameliorate the social and economic burden of COVID-19. This daunting healthcare demands require a well-motivated and satisfied workforce despite the scare resources and poor remunerations that can create employee burnout [1].

Long before COVID-19, Africa had its own challenges with availability and quality of health professionals but the situation has worsened with the global pandemic. Health professionals in Africa often lack motivation as results of high workload, poor remuneration, poor working environment, discrimination etc [2]. Ghana is one of the African countries that face severe challenges with health professionals. In 2019, Ghana was added to the list of countries with severe health professional challenges by the World Health Organization (WHO). The WHO further predicted that the persistence of the challenge can significantly affect quality health service delivery [3]. This is because Ghana has one of the lowest health worker-to-population ratio in the whole world. Poor job satisfaction and lack of managerial support is largely responsible for the brain drain of health workers from Ghana and other African countries to developed countries [4] Within Ghana, health professionals persistently move from the poor rural to urban areas causing severe challenges to rural health service delivery [5]. This may explain why Ghana is among the countries with high infection and mortality of COVID-19 in Africa [6]

For decades, researchers have investigated the drivers of employee motivation and wellbeing, yet concrete solutions continue to elude even the most developed medical system in Africa. Bakker and Demerouti’s [7] job demands-resources (JD-R) model is one of the models often used to explain the drivers of employee engagement, burnout, satisfaction and performance in the work place. This model assumes that the source employee wellbeing or satisfaction is job or occupation specific but the sources can be categorized into job demands factors and job resource factors. Bakker and Demerouti [7] argue that high job demand causes both mental and physical exhaustion which leads to depletion of energy and health problems for employees or health impairment. Conversely, job resources help to foster engagement and stimulate higher role performance. When employees have adequate job resources, it buffers the impact of job demand on stress, thus confirming the motivation potential of availability of job resources.

Unfortunately, in the context of hospitals in Africa, both job demand factors and job resources factors remain a major problem that contemporaneously influences dissatisfaction and performance [8]. For this reason, health professionals in Africa often rely on other sources of motivation to nourish their psychological, emotional and mental wellbeing and satisfaction for the work they do. Job autonomy and Luthans et al.’s [9] psychological capital theory (PsyCap) provides a good indicator of the source of employee motivation and satisfaction in the context of hospital employees in Africa as a whole and Ghana in particular. Luthans et al. [9] explains psychological capital as a person’s positive psychological state of development that enables him to develop strong hope, self-efficacy, resilience and optimism in the pursuit of work. Luthans et al. [10] further contends that PsyCap answers the question of who one is which eventually gives impetus to what he or she wants to achieve. Hope—as a component of psychological capital—refers to an individual’s motivation to succeed at a specific task in a set context and the way or means by which that task may be accomplished, in other words, the goals, pathways and agency to completing a given task [11].

On the other hand, the concept of optimism refers to a person’s expectation of a positive outcome [12] whereas the resilience component of psychological capital refers to a person’s ability to bounce back from adversity, uncertainty, risk or failure, and adapt to changing and stressful life demands [13,14]. Finally, the self-efficacy aspect of psychological capital includes an individual’s confidence in his capability to mobilize motivation, cognitive resources and courses of action to achieve high levels of performance [15]. According to Luthan et al. [16] positive psychological capital facilitates the individual’s motivational predispositions through cognitive resources that also help the individual to experience rewards from the present moment as well as raising future fortunes.

Since the work of Luthan et al. (2005), several researchers have applied the psychological capital theory to explain behavioral patterns among different employee groups. For example, a study conducted by Lu et al. [17] found that psychological capital was positively related to job satisfaction and also found to predict job performance. The study noted that medical doctors who showed higher-order levels of psychological capital were confident in their abilities and executed their tasks successfully. Psychological capital and trust help to change the performance of individual performance to job satisfaction [18]. Previous studies have focused on the interplay between motivation and job satisfaction as the panacea to high performance in the healthcare sector where productivity is not easy to measure. Job satisfaction ensures that hospital employees’ attitudes have very good impressions about the work they do in order to make total impact on the job. Job satisfaction comes from employees’ positive psychology, motivation, efficiency, and positive mood, which are urgently needed, in a resource-depleted healthcare system in Ghana. Moreover, Asiamah and Mensah [19] argues that job satisfaction is negatively associated with nurses’ turnover intention which is indispensable qualities needed from hospital professionals in Ghana in the midst of the debilitating effect of numerous pandemic and epidemic that continues to plague the continent.

Psychological capital has also been linked to employee’s autonomy. Similarly, employee autonomy has been linked to satisfaction and performance. Hackman and Oldham [20] define autonomy as the degree to which employees have control and discretion over how to carry out their duties. Virick et al. [21] also defines perceived autonomy as the degree to which an individual, in accordance with his or her own free conduct, is not subject to external interference in a given situation and considers his or her conduct. Cai et al. [22] theorized and examined how supervisor support for creativity or autonomy effectively activate the psychological capital associated with self-reported employee creativity. The authors concluded that PsyCap is most effective at enhancing creativity when both autonomy and job characteristics are high. Based on a study of 403 bank employees in Bangkok, Narumol et al. [23], found out that positive psychological capital and job autonomy interact to generate intrinsic work motivation that stimulates higher employee performance. A more recent study by Li et al. [24] complements the intricate relationship between psychological capital, job autonomy and other positive work outcomes. The study noted that psychological capital partially mediates the relationship between humorous leadership and employee workplace creativity. The authors further asserted work autonomy significantly moderates the relationship between employee’s psychological capital and employee creativity. The self-determination theory enforces the importance of job autonomy in these interactions by indicating that when an employee feels that his or her decision sourcing or acting is self-determining, the individual becomes intrinsically motivated as posited in the self-determination theory [25]

Another study that explores the importance of job autonomy in stimulating positive work outcomes is the work of Asiabar et al. [26]. They explored the proximal and distal consequences of telecommuting (psychological control or perceived autonomy) on work and family life. The results showed that job autonomy influenced several employee work attitudes and positive work outcomes such as performance, job satisfaction and turnover. Similarly, the job-characteristics model (JCM) has been used by Goldman, and Tabak [27] to establish the relationship between autonomy of telecommuting employees and their job satisfaction. Complementing and extending prior studies, Jung [28] found out that remote staff exhibited higher perception of autonomy which translated into strong organizational commitment and loyalty.

Examining the current literature, it is evident that while the relationship between job autonomy and other attitudes and outcomes have been investigated, the generalizability of the interaction between psychological capital and job autonomy to achieve higher satisfaction among healthcare professionals is barely addressed and remains a major gap that need to be addressed. Our work is particularly inspired by the earlier studies such as Burcharth et al. [29] that argue that employee autonomy has the potential to mediate a wide range of positive employee outcomes such as innovation capability of employees. Modern healthcare systems require innovative employees instead of routine workers. Similarly the study is inspired by Katerndahl et al. [30] who argues that psychological autonomy can positively mediate contributes to desirable employee behaviors such as competence, work-life balance and well-being which are important attributes in the hospital. Identify the extent to which satisfaction can be procured through psychological autonomy at the hospitals as this study seeks to achieve will be an important first step for advanced researchers to build on in the future.

This research fills this gap by empanelling and ensemble of more sophisticated mixed structural equation model to test the role of job autonomy for strengthening the direct effect of psychological capital on job satisfaction. The structural equation model is preferred as it guarantees robustness of inference compared with the traditional regression models that are dominant in the stock of current literature that have explored related subjects. After discussing the methodology for this research in the next section, the results are subsequently presented in the following section. Finally we discuss the findings, present the limitations and outline future research direction.

## 2. Materials and Methods

To achieve the objectives of the research, a quantitative research method based on survey research strategy was employed to collect data from qualified respondents in Ghana’s healthcare sector. As the study was based on individual experiences of healthcare professionals, the sample was procured from respondents who were selected randomly from the Korle-Bu Teaching Hospital, the Komfo Anokye Teaching Hospital, the Tamale Teaching Hospital, the Cape Coast Teaching Hospital and the Ho Teaching Hospital. These five hospitals are the main tertiary level hospitals in Ghana. In addition, data was collected from health professionals working at the 37 military hospitals. This institution is not a tertiary hospital but a very important military hospital in West Africa. It is the main referral hospital for national and regional emergency health services by the United Nations, the Red Cross and other international organizations.

Additionally, the six hospitals were chosen because they receive the highest inflow of patients on a daily basis and their staff are more susceptible to emotional and psychological crisis due to work load, work schedules and poor conditions of services that are generally low in a developing sub-Saharan country such as Ghana. Moreover, recent strike actions and media complaints about poor working conditions and resignations by dissatisfied healthcare professions have largely emanated from workers in these main hospitals in Ghana.

Further, even though clinicians are considered premium staff in healthcare facilities; the hospital work is interdependent on both clinical and non-clinical staff. Doctors cannot work in the absence of nurses, laboratory technicians, hospital administrators, and other allied health workers. Thus, these categories of healthcare workers are subject to similar workload, work schedule but different conditions of service. In the case of non-clinicians, their service conditions are far below their clinical counterparts, hence, they show more dissatisfaction. To that extent these healthcare workers often lose the needed to work in a difficult work environment such as a hospital and may occasionally require reassurance of managerial psychology to reboot their enthusiasm. An initial number of 400 respondents (both clinical and non-clinical staff) were selected, contacted and agreed to participate in the research but only 385 questionnaires were actually returned. This represents nearly 95% successful rate. Table 1 shows the total number of clinical and non-clinical staff sampled in each of the hospitals.

A questionnaire data collection instrument was designed based on indicators that have been previously used to carry out similar work. It was self-administered to the respondents between March 2019 and December 2019. Respondents signed a consent form before participating in the research. Prior to administering the questionnaires, it was pre-tested on an initial sample (21 respondents) that did not form part of the final sample but within the population.

The results from the pre-test sample were used to reword the questionnaire and fine-tune other parts to improve their reliability. As the study involved a human sample, written ethical approval was obtained from the Jiangsu University Research Ethic Committee and the Ghana’s Ministry of health Research Ethics Committee before carrying out the data collection. A closed-ended style of response was used to design the questionnaire. This means that each question had alternative responses from which respondents could make a choice. Using this approach helped to speed up the data collection since the respondents work in a very fast moving environment and had no much time. Secondly, a closed ended approach was used because it can help to improve the coding of the data. There were six constructs in all namely psychological capital (hope, optimism, resilience, and self-efficacy) and job autonomy and job satisfaction. These were coded as follows:

### 2.1. Psychological Capital and Job Autonomy

Positive psychological capital or simply psychological capital has been conceptually identified as consisting of four positive psychological resources of self-efficacy, hope, optimism, and resilience [10]. According to Luthans, et al. [31] psychological capital has been established to give competitive advantage to firms or institutions through the development of people’s hope, optimism, resilience and self-efficacy. The level of freedom employees experience in terms of decisions making at the workplace is referred to as job autonomy. Experiencing job autonomy is seen as a factor that promotes work [32]. Saragih et al. [33] posits that there is a lot of creativity and accomplishment of tasks when employees experience high job autonomy when their psychological capital is well developed. Further, it has been established that employees who experience job autonomy are well encouraged which pushes them to go beyond their limit to get the best out of them [34]. It is hypothesized from the above that:

**Hypothesis** **1a** **(H1a):** *hope will positively influence job autonomy;*

**Hypothesis** **1b** **(H1b):** *self-efficacy will positively influence job autonomy;*

**Hypothesis** **1c** **(H1c):** *resilience will positively influence job autonomy;*

**Hypothesis** **1d** **(H1d):** *optimism will positively influence job autonomy.*

### 2.2. Job Autonomy and Job Satisfaction

There are various determinants of employee job satisfaction and one of them is job autonomy. Hackman and Oldham [20] defined job autonomy as “the degree to which the job provides substantial freedom, independence, and discretion to the employee in scheduling the work and in determining the procedures to be used in carrying it out” (p. 162). The linkage between job autonomy and job satisfaction can best be explained using the JD-R model. In this model, the jobs people engaged in are divided into job demands and job resources. While job demands concern itself with the cost in physiology, social, psychological, or organizational sides of the job like emotional demands, job resources lessen the impact of job demands and its costs to stimulate some level of learning, growth and development [7].

In the JD-R model, job autonomy is aligned with job resource which seeks to prevent the negative impact job demands will bring. Therefore, absence of job autonomy raises the negativities of absenteeism, stress, repetitive strain, and ill health whereas presence of job autonomy leads to higher employee job satisfaction [7]. Many researches have established the important relationship job satisfaction has with job autonomy. Taylor et al. [35] found that job satisfaction can be explained by autonomy to a great extent.

The conclusions by researchers on the influence of job autonomy on job satisfaction has been quite consistent, the findings have been mostly that job autonomy leads to job satisfaction [35,36] on the same assumption, a lack of autonomy will result in higher levels of stress which in turn can lead to dissatisfaction in one’s work [7]. It is postulated that:

**Hypothesis** **2** **(H2):** *Job autonomy will positively influence employees’ job satisfaction.*

### 2.3. Measurement of Psychological Capital

**Psychological Capital:** PsyCap was measured using Luthans et al.’s [31] psychological capital questionnaire (PCQ). With this scale, PsyCap is conceptually identified as consisting of four positive psychological resources of self-efficacy, hope, optimism, and resilience. There are 24 questions in this scale and are calibrated on a six-point Likert scale measure. The responses range from 1 (strongly disagree) to 6 (strongly agree).

**Measurement of Autonomy:** The self-determination theory’ questionnaire proposed by Deci and Ryan [37] was used to measure the autonomy construct. This questionnaire uses a five point Likert scale to calibrate the responses. A strongly agree responses is denoted with the value 5 whereas a strongly disagree responses is denoted with the value 1. Examples of some of the questions on this scale include; “I feel a sense of choice and freedom in the things I undertake”, “I feel that my decision reflects what I really want”. This questionnaire has also been validated in current research studies such as [3,8].

**Measurement of Job Satisfaction:** Job satisfaction was measured with the Minnesota Satisfaction Questionnaire (MSQ) developed by the University of Minnesota. The responses are also ranked on a five point Likert scale where 1 denotes very dissatisfied and 5 denotes very satisfied. The MSQ has two versions namely a shorter version and a longer version. This study applied the short version. The short form of the MSQ can be seen as a composite of a number of job facets. Scores are created by summing items so as to show each participant’s satisfaction level ranging from 20 to 100. A score of 60 would indicate moderate, a score ranging from 61 to 79 would indicate ‘moderate to not fully satisfied’ and a score of 80 and above would indicate satisfied. This questionnaire has been widely used in measuring employee job satisfaction in contemporary studies with robustness of inference [38,39].

### 2.4. Data Analysis

To test the hypotheses, a structural equation regression model was constructed based on the conceptual framework presented in the earlier sections of the research. Descriptive statistics was first computed to determine the mean, standard deviation and skewness of the constructs. The latter is necessary in determining the normality of data to determine the type of inferential analysis to carry out. Based on the results a Pearson product moment correlation coefficient was computed to determine the existence of possible multicolinearity among the variables. A mixed structural equation model was then constructed to establish the relationship between psychological capital, job autonomy and employee job satisfaction among the selected respondents. Using a structural equation model was preferred to the traditional regression model since it produces more robust outcome. It is the only known statistical procedure that is able to determine the coefficient of regression in a multivariate regression as if these multiple various acted independently. All data analysis was done by SPSS for Windows Ver. 22.0, and AMOS version 22 software and a two-tailed probability value of <0.05 was considered to be statistically significant.

## 3. Results

### 3.1. Descriptive Analysis

The descriptive statistics of the study is presented in Table 2. It shows the percentage of respondents in terms of their age groups, gender, educational level and number of years of service. This information was helpful in conducting the analysis of variance of responses among different groups of respondents. The information in Table 3 on the other hand shows the results of the analysis of variance of the variables of study in relations to age groups. It sought to evaluate whether different age groups responded differently to each of the variables of study. At a 95% confidence interval, a significant value in excess of 0.05 means that no difference is observed in the age groups. This is the case with Table 3 because each of the significant values for hope, optimism, self-efficacy, resilience, autonomy and satisfaction were higher than 0.05.

The information in Table 4 is also an ANOVA test results but the objective is to determine whether men and women demonstrated significant differences in their response to questions about hope, optimism, self-efficacy, resilience, autonomy and satisfaction. In this case, the analysis also shows that the significant values of each of the constructs are above 0.05, which means that generally the differences among male and female respondents were not statistically significant at the 95% confidence interval and could not be relied upon to deduce conclusions for the research.

Finally differences in the type of work that one does can affect the indicants of psychological capital, job autonomy and satisfaction. This is particularly important in the case of hospitals. The analysis of variance presented in Table 5 shows the observed differences and their significant values regarding how different groups of employees answered questions pertaining to hope, optimism, resilience, self-efficacy, autonomy and job satisfaction. In this case, the results also show high significant values in excess of 0.05, indicating that the statistical significance of differences length type of work is also insignificant at the 95% confidence interval.

### 3.2. Measurement Model

Table 6 shows the factor loadings extracted from the correlation between the reflective and the formative variables. As a rule of thumb, reflective variables must have loadings of at least 0.7. The table shows that 1 item of hope, 2 items of optimism, 1 item of resilience and 2 items of self-efficacy did not meet the required threshold. They were, however, kept in the work to maintain the content validity of the scale. As such, modification indices were consulted and the factors were accepted because loadings are significant and the values are slightly below the critical level. The table also shows the final set of admissible loadings and items that qualified for further analysis.

Table 7 shows the Kaiser-Meyer-Olkin (KMO) sampling adequacy test and Bartlett’s sphericity test. The results of the KMO and Bartlett’s test returned a KMO value of 0.829 and a significant Bartlett’s test value of 0.000. Typically, a KMO value greater than 0.5 is the minimum acceptable value (Kaiser, 1974), while a value between 0.7 and 0.8 is a highly acceptable value. Kaiser further asserts that a value above 0.9 is excellent, indicating that the sample is sufficient for further analysis. Similarly, all the communalities were sufficiently high (all of them were above 0.0 and most of them were above 0.6). This indicates that the selected items were adequately correlated for factor analysis. The reproduced correlation matrix showed only 2% non-redundant residuals greater than 0.05 and this further confirms the adequacy of the variables and the 6 factor model.

**Reliability**: Table 8, on the other hand, shows the Cronbach alpha coefficients of the composite values. All measures show adequate levels of reliability as they outperformed the threshold value of 0.7. The factors are all reflective, their indicators are highly correlated and are largely interchangeable (Jarvis, MacKenzie, & Podsakoff, 2003).

### 3.3. Validity

Regarding validity, the factors demonstrate sufficient convergent validity, since all the loadings were all above 0.350 which is recommended as the minimum threshold if the sample size is 300 [40]. The factors also demonstrate sufficient discriminant validity, as the correlation matrix shows no correlations above 0.700, and there are no problematic cross-loadings. This six-factor model had a total variance explained of 60%, with all extracted factors having Eigen values above 1.0 except one, which was close at 0.989.

Table 9 shows the descriptive statistics for both the reflective the formative variables. The results of the mean, standard deviation and skewness have been presented. Normality was confirmed using the Kolmogorov Smirnov–Shapiro Wilks test values [41]. Subsequently, the Pearson’s product moment correlation test was used to establish to determine multicolinearity as shown in the inter correlation matrix in Table 9. The results show that the correlation values among the independent variables are below the 0.5 threshold, which indicates low correlation or an absence of multicolinearity among the reflective variables. The variance inflation factor (VIF) was further computed to confirm absence of multicolinearity among the formative variables. The rule of thumb is that variance inflation factor: 1 = not correlated. Between 1 and 5 = moderately correlated. Greater than 5 = highly correlated. The results show that all the VIF values are below 0.5 and support the absence of multicolinearity among the reflective variables.

### 3.4. Confirmatory Factor Analysis (CFA)

Following this, a confirmatory factor analysis was conducted. This is to establish the degree to which the model fits the hypothesized structural equation measurement model. The results in the goodness of fit and the model fit measures table were used to determine the fitness of the model. The results presented in Table 10 shows that the goodness of fits for the measurement model is sufficient Table 10:

Table 11, on the other hand, shows the output of the model fit measures. Firstly, a composite reliability measure of internal consistency is presented. The result is in excess of 0.7 for all constructs. This further supports the Cronbach’s alpha’s coefficient’s values of high level of internal consistency among the composite items. As a result, the lower indicator reliability of composite reliability (CR) can be accepted.

Similarly, the results show that convergent validity values are acceptable since all the factor loadings exceed the 0.6 threshold. Again, for all the factors, the average variances extracted (AVE) value was above 0.50, as indicated in Table 11, with the exception of autonomy, which had a close value of 0.495. However, as this factor minimally correlates with the other factors in the model, and considering that it obtained a reliability score and has CR value of 0.799 (above the 0.700 threshold), it was considered admissible. This decision is supported in Pallant [40] who argues that constructs that may not be internally strong but at least reliable and distinct within a model can be admitted with close to 0.5 AVE. As noted in Larcker [41], the square root of AVE of each latent variable is a good predictor of discriminant validity if the value is larger than other correlations values among the latent variables. Table 11 also shows the square roots of average variances extracted (AVE) along the diagonal line in bold numbers. This, again, indicates that discriminant validity is well established in the model.

### 3.5. Path Analysis

**Figure 1:**
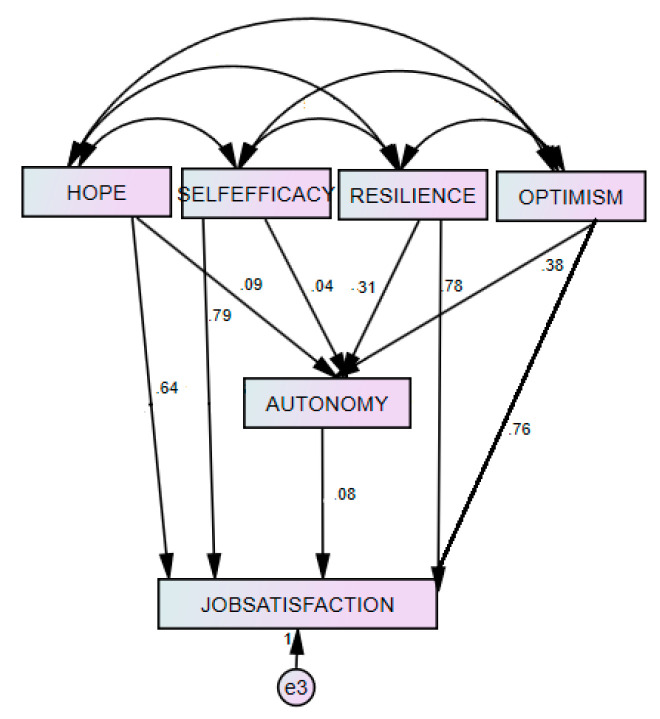
Structural path analysis.

Finally, a structural path analysis (Figure 1) was conducted to test the significance or otherwise of the various hypotheses and details are presented in Table 12.

In Table 12, the path coefficient value confirms a positive relationship between self-efficacy and job autonomy (β = 0.04). However, this relationship is not significant *p* > 0.05 hence Hypothesis 1b must be rejected. Beside all path coefficients from the other psychological capital constructs; hope (β = 0.09), resilience (β = 0.31); and self-efficacy (β = 0.04) to job autonomy were positive and statistically significant. This implies that Hypotheses 1a (H1a), 1c (H1c) and 1d (H1d) must be accepted. Finally the relationship between autonomy and job satisfaction is also positive and statistically significant. The β = 0.08 and the *p* value is 0.024 which is statistically significant. The model also show that PsyCap explains 65% of employee job autonomy need with an R^2^ = 0.651. According to Iliopoulou and While [42] the mediating effect is determined when three conditions are met. Firstly, there should be a pre-existing relationship between the dependent variables and the mediating factor. Secondly, there should be a pre-existing direct relationship between the mediating factor and the dependent variable. Finally, the effect of the independent variable on the dependent variable should be lower than the direct relationship in the presence of the mediating factor. The results further confirm that job autonomy mediates the relationship between psychological contract indicants such as hope, resilience and optimism and job satisfaction.

## 4. Discussions and Conclusions

In the extant literature, employee job satisfaction is well established as a major factor to stimulate positive organizational behaviors. A major source of positive organization behavior is that healthcare professionals resist control but desire some degree of autonomy as a basic psychological need. Throughout the extant literature, numerous factors have been listed as important drivers of employees’ job autonomy. This study focused on the validity in the extent to which psychological contract attributes can influence employee’s autonomy to promote job satisfaction. Data was collected from 385 health professionals in Ghana and a structural equation model was applied to analyze the relationships. The results show a positive relationship between hope and job autonomy. Similarly, optimism and self-efficacy largely influences job autonomy of the healthcare professionals. The only exception was resilience which recorded a positive but insignificant effect on employee’s autonomy. The findings implies that a relationship exist between psychological capital and job autonomy which translates into job satisfaction among health professionals in Ghanaian hospitals [43]. The results further confirm that job autonomy mediates the relationship between psychological contract indicants such as hope, resilience and optimism and job satisfaction.

The results of this study affirm several findings in the previous literature. Job autonomy has been found to be linearly related to job satisfaction relative to other professions [44,45,46,47]. Similarly, Iliopoulou and While [43] found out that overall, nurses reported acting moderately autonomously. They argued that professional autonomy is generally considered a highly desirable nursing attribute and a major factor in nurse job satisfaction. In the critical care environment, a high level of accountability, responsibility and autonomy are required to optimize outcomes of critically unstable patients. In most public institutions, the relationship between leaders and their followers is mostly determined by the traditions and structure of the organization, those organizations that follow procedures do not have lot of this autonomy, however, flatter organizations use autonomy, empowerment, and participation to succeed [48].

The level of autonomy that employee exercises or enjoys break the gap between higher and lower authority in the organization, gives people the independence to perform their duty. When employees exercise greater professional autonomy, they become more creative and innovative, generate new ideas and solve problems more easily. Professional autonomy helps employees to build confidence and develop quality relationships among employees and superiors based on trust. Most importantly, greater autonomy strengthens job satisfaction. An increased in autonomy reduces the level of discontent since individual employees have different psychological needs in their area of work.

## 5. Future Research and Limitations

Other aspect of this research can be conducted to broaden the scope of knowledge presented in this paper. Today as never before, COVID-19 and its threat to frontline health workers has reignited the call for greater safety for health professionals. To date more than 10,000 hospital workers have been killed by COVID-19 across the globe. Future research must investigate the impact of health-related safety concerns on psychological and mental stress and satisfaction of nurses. Similarly, specialized groups such as family doctors or general practitioners have unique working environment and the impact of COVID-19 on family practice must be studied in detail. This study has limitations. The sample was randomly selected from six hospitals and there is a potential sampling bias despite all possible caution. The respondents could have been dishonest in their responses, even though they were informed to be as frank as possible. Thirdly, the case in Ghana may not be exactly the case in other countries, hence, cultural and geographical factors limit the generalizability of this paper.

## Figures and Tables

**Table 1:** Profile of respondents from respective hospitals.

	Clinical Staff		Non-Clinical Staff	
	Population	Sample	Population	Sample
Korle Bu Teaching Hospital	1113	40	2071	45
Komfo Anokye Teaching Hospital	1083	36	1985	47
Cape Coast Teaching Hospital	523	38	621	29
Ho Teaching Hospital	325	27	402	27
Tamale Teaching Hospital	489	28	592	23
37 military hospitals	302	31	398	29

**Table 2:** Characteristics of the respondents of the study (*n* = 385).

Demographic Variable	Number	Percentage
**Gender**		
Male	126	32%
Female	259	65%
**Age Group**		
20–30	131	33%
31–40	136	34%
41–50	97	24%
51+	21	5%
**Educational Level**		
Middle	27	7%
Diploma	153	38%
Degree	133	33%
Masters	72	18%
**Years of Service**		
1–10 Years	220	55%
11–20 Years	140	35%
20+ Years	25	6%

Table 2 shows that females constituted 67.3% of the sample and males comprised of 32.7%. Ages spanning from 31–40 dominated the sample with 35.3% with the ages 51+ with just 5.4% of the total population sampled. The study sample had more of a younger population with 57.1% who had worked for a period of 1–10 years.

**Table 3:** Results of ANOVA test of difference of variables between age groups.

ANOVA						
		Sum of Squares	df	Mean Square	F	Sig.
Hope	Between Groups	18.592	1	18.592	11.448	0.101
	Within Groups	1425.952	878	1.624		
	Total	1444.544	879			
Optimism	Between Groups	105.464	1	105.464	87.745	0.520
	Within Groups	1055.299	878	1.202		
	Total	1160.762	879			
Self-Efficacy	Between Groups	56.006	1	56.006	81.006	0.063
	Within Groups	607.038	878	0.691		
	Total	663.044	879			
Resilience	Between Groups	0.407	1	0.407	0.350	0.554
	Within Groups	1022.120	878	1.164		
	Total	1022.527	879			
Autonomy	Between Groups	5.655	1	5.655	5.854	0.096
	Within Groups	848.108	878	0.966		
	Total	853.762	879			
Satisfaction	Between Groups	2.005	1	2.005	2.709	0.100
	Within Groups	649.954	878	0.740		
	Total	651.959	879			

**Table 4:** Results of ANOVA test of difference of variables between male and female.

ANOVA						
		Sum of Squares	df	Mean Square	F	Sig.
Hope	Between Groups	26.544	2	13.272	8.208	0.080
	Within Groups	1418.000	877	1.617		
	Total	1444.544	879			
Optimism	Between Groups	76.239	2	38.120	30.826	0.120
	Within Groups	1084.523	877	1.237		
	Total	1160.763	879			
Self-Efficacy	Between Groups	203.508	2	101.754	194.191	0.061
	Within Groups	459.537	877	0.524		
	Total	663.044	879			
Resilience	Between Groups	415.204	2	207.602	299.786	0.073
	Within Groups	607.323	877	0.693		
	Total	1022.527	879			
Autonomy	Between Groups	118.911	2	59.455	70.956	0.190
	Within Groups	734.852	877	0.838		
	Total	853.763	879			
Satisfaction	Between Groups	52.734	2	26.367	38.590	0.630
	Within Groups	599.225	877	0.683		
	Total	651.959	879			

**Table 5:** Results of ANOVA test of difference of variables between different occupations.

ANOVA						
		Sum of Squares	df	Mean Square	F	Sig.
Hope	Between Groups	517.083	4	129.271	121.959	0.060
	Within Groups	927.461	875	1.060		
	Total	1444.544	879			
Optimism	Between Groups	218.658	4	54.664	50.771	0.110
	Within Groups	942.105	875	1.077		
	Total	1160.762	879			
Self-Efficacy	Between Groups	30.175	4	7.544	10.430	0.065
	Within Groups	632.869	875	0.723		
	Total	663.044	879			
Resilience	Between Groups	300.396	4	75.099	90.997	0.057
	Within Groups	722.131	875	0.825		
	Total	1022.527	879			
Autonomy	Between Groups	160.213	4	40.053	50.532	0.109
	Within Groups	693.550	875	0.793		
	Total	853.763	879			
Satisfaction	Between Groups	8.289	4	2.072	2.817	0.084
	Within Groups	643.670	875	0.736		
	Total	651.959	879			

**Table 6:** Factor loadings.

Pattern Matrix ^a^						
	Factor					
	1	2	3	4	5	6
Hope1	0.806					
Hope2	0.757					
Hope3	0.711					
Hope4	0.949					
Hope5	0.739					
Hope6	0.697					
Optimism1		0.836				
Optimism2		0.829				
Optimism3		0.776				
Optimism4		0.775				
Optimism5		0.692				
Optimism6		0.651				
Self-Efficacy1			0.881			
Self-Efficacy2			0.771			
Self-Efficacy3			0.767			
Self-Efficacy4			0.857			
Self-Efficacy5			0.674			
Self-Efficacy6			0.689			
Resilience1				0.758		
Resilience2				0.896		
Resilience3				0.863		
Resilience4				0.819		
Resilience5				0.701		
Resilience6				0.691		
Autonomy1					0.918	
Autonomy2					0.748	
Autonomy3					0.839	
Autonomy4					0.743	
Satisfaction						0.769
Satisfaction						0.843

Extraction method: maximum likelihood. Rotation method: Promax with Kaiser normalization. ^a^ Rotation converged in 5 iterations.

**Table 7:** Sampling adequacy test.

Kaiser-Meyer-Olkin Measure of Sampling Adequacy.		0.829
Bartlett’s Test of Sphericity	Approx. Chi-Square	303.746
	df	382
	Sig.	0.000

**Table 8:** Construct reliability measures.

Latent Variables	Cronbach’s Alpha Coefficients	Specification
Hope	0.846	Reflective
Self-Efficacy	0.866	Reflective
Resilience	0.836	Reflective
Optimism	0.843	Reflective
Autonomy Need	0.913	Reflective
Job Satisfaction	0.875	Formative

**Table 9:** Descriptive statistics.

Variable	Mean	SD	Skew	Shapiro Wilks	1	2	3	4	5	6
Hope	4.2	0.03	0.38	0.54	1					
Optimism	4.6	0.13	0.06	0.63	0.24	1				
Resilience	5	0.08	0.29	0.72	0.37	0.14	1			
Self-Efficacy	4.3	0.61	0.09	0.59	0.20	0.49	0.03	1		
Autonomy	4	0.32	0.43	0.63	0.38	0.20	0.04	0.32	1	
Job Satisfaction	3.9	0.96	0.27	0.57	0.54	0.49	0.31	0.73	0.69	1
VIF					1.12	1.61	1.43	1.02	1.09	1.47

**Table 10:** Goodness of fit indexes.

Measure	Estimate	Threshold	Interpretation
CMIN	489.91	--	--
DF	392	--	--
CMIN/DF	1.459	Between 1 and 3	Excellent
CFI	0.983	>0.95	Excellent
SRMR	0.031	<0.08	Excellent
RMSEA	0.032	<0.06	Excellent
PClose	0.975	>0.05	Excellent
Cutoff Criteria			
Measure	Terrible	Acceptable	Excellent
CMIN/DF	>5	>3	>1
CFI	<0.90	<0.95	>0.95
SRMR	>0.10	>0.08	<0.08
RMSEA	>0.08	>0.06	<0.06
PClose	<0.01	<0.05	>0.05

**Table 11:** Model fit measures.

	CR	AVE	MSV	1	2	3	4	5	6
Hope	0.829	0.648	0.002	0.792					
Optimism	0.863	0.491	0.488	0.043	0.690				
Resilience	0.883	0.695	0.169	0.042	0.051	0.822			
Self-Efficacy	0.844	0.615	0.169	0.035	0.004	0.404	0.772		
Autonomy	0.799	0.495	0.181	0.022	0.419	0.037	0.016	0.675	
Job Satisfaction	0.736	0.501	0.488	0.001	0.688	0.079	0.041	0.393	0.697

**Table 12:** Regression weights.

				Estimate	
Paths				Unstandardized	*p*-Value
AT	<---	Hope	b1	0.09 *	0.012
AT	<---	Optimism	b3	0.78 *	0.023
AT	<---	Resilience	b5	0.31 *	0.003
AT	<---	Self-Efficacy	b2	0.04 *	0.061
JS	<---	Hope	b1a	0.64 *	0.010
JS	<---	Optimism	b3a	0.76 *	0.002
JS	<---	Resilience	b5a	0.78 *	0.000
JS	<---	Self-Efficacy	b2a	0.79 *	0.051
JS	<---	Autonomy	b4a	0.08 *	0.024

* *p* < 0.05.
